# Luteinizing Hormone/Human Chorionic Gonadotropin Receptor Immunohistochemical Score Associated with Poor Prognosis in Endometrial Cancer Patients

**DOI:** 10.1155/2018/1618056

**Published:** 2018-04-02

**Authors:** Flavia Sorbi, Elisabetta Projetto, Irene Turrini, Gianna Baroni, Serena Pillozzi, Viola Ghizzoni, Federica Vergoni, Francesca Castiglione, Francesca Malentacchi, Massimiliano Fambrini, Ivo Noci

**Affiliations:** ^1^Department of Biomedical Clinical and Experimental Sciences, University of Florence, Viale Morgagni 50, 50134 Florence, Italy; ^2^Division of Pathological Anatomy, Department of Surgery and Translational Medicine, University of Florence, Largo Brambilla 3, 50134 Florence, Italy; ^3^Department of Experimental and Clinical Medicine, University of Florence, Viale Morgagni 50, 50134 Florence, Italy

## Abstract

The aim of this study was to develop a scoring system of the immunohistochemical (IHC) expression of luteinizing hormone/human chorionic gonadotropin receptor (LHCG-R) in endometrial cancer (EC) patients. Nonconsecutive hysterectomy specimens containing EC collected from April 2013 to October 2015 were selected. Hematoxylin-eosin stained sections from each case were reviewed and representative sections from each tumor were selected. IHC staining was performed for the detection of LHCG-R. The percentage of stained cells and the staining intensity were assessed in order to develop an immunohistochemical score. Moreover, we examined the correlation of the score with grading and lymphovascular space invasion (LVSI). There was a statistically significant positive correlation between grading and IHC scoring (*p* = 0.01) and a statistically significant positive correlation between LVSI and IHC score (*p* < 0.01). In conclusion, we suggest that the immunohistochemical score presented here could be used as a marker of bad prognosis of EC patients. Nevertheless, further studies are needed in order to validate it. The study was registered in the Careggi Hospital public trials registry with the following number: 2013/0011391.

## 1. Introduction

Endometrial cancer (EC) is the most common gynecological tumor. The incidence of endometrial cancer is going to increase in the upcoming years. [1]. Most patients are diagnosed at stage I and the 5-year overall survival ranges from 74% to 91% [1]. Conversely, patients with metastatic disease have a median survival of 7–12 months, in spite of treatment [2].

In order to classify for improving the EC prognosis, in 1983, Bokhman described two pathogenetic types of endometrial carcinomas characterized by different metabolic, morphological, and endocrine profiles: type 1 is more common (~70–80%) and consists of endometrioid histology and is low grade, diploid, hormone-receptor positive and typical of obese women, and type 2 (20–30%) consists of nonendometrioid histology, is high-grade, aneuploid, poorly differentiated, hormone-receptor negative and typical of nonobese women, and is associated with higher risk of metastasis and poor prognosis. [3].

Nowadays, FIGO stage (International Federation of Gynecology and Obstetrics), tumor histotype, depth of myometrial invasion, presence of lymphovascular space invasion (LVSI), and histological grading are used to tailor treatment and to predict prognosis [1]. However, this management may lead to undertreatment [4].

Therefore, a variety of molecular biomarkers are under investigation such as PI3 K/PTEN/AKT/mTOR pathway alterations, CTNNB1, KRAS, and TP53 mutations, and methylation profile of MLH1 promoter [2] in order to improve the detection of women with increased risk of metastasis and local recurrence and consequently tailor treatments according to the patient's molecular profile.

The most comprehensive molecular study of ECs has been performed by The Cancer Genome Atlas (TCGA) network, based on integrated genomic (whole genome sequencing, exome sequencing, microsatellite instability (MSI) evaluation, and copy number analysis), transcriptomic, and proteomic analysis, suggesting a new classification in 4 different classes depending on genomic features that may suggest appropriate and personalized treatments [5].

The presence of luteinizing hormone/human chorionic gonadotropin receptor (LHCG-R) in EC has already been described in previous studies [6–10], suggesting a key role in cell line proliferation an in invasion in vitro and in preclinical models. Our group demonstrated that LHCG-R acts through its receptor LH-R on the recruitment of protein kinase A (PKA), which induced the activation of beta 1 integrin receptors and the secretion of active matrix metalloproteinase-2 ending in the triggering of cell invasiveness [6, 7]. Further we investigated the role of LHCG-R in preclinical models showing that its overexpression increased the ability of EC cells in local invasion and metastatic spread in orthotopic xenograft mice [8]. Moreover, we reported that a patient treated primarily with LHRH analogue showed no clinical progression of the disease, giving new evidence of the favourable impact of LHRH analogue treatment [9]. These “in vivo” and preclinical data suggested a key role of LHGC-R as bad prognostic marker; moreover they suggested that LHCG-R could be used for the management of surgical and postsurgical treatment and for surveillance.

In the literature, nowadays, there have been no prospective clinical studies that can confirm the role of LHCG-R in patients with EC. However, studies of this type need to have a simple, cost-effective and easily replicable system for DETERMINING LHCG-R in tumor tissue. Our previous studies evaluated LHCG-R expression by RT-qPCR [7, 9]. Instead, in our latest study, we also applied immunohistochemical (IHC) evaluation [10]. The IHC technique is widely used in oncology diagnostics, both for gynecological and nongynecological specimens, and meets the above requirements of simplicity, affordability, and repeatability [11, 12].

The aim of this study was to develop a scoring system for the presence and the amount evaluation of LHCG-R by IHC in EC. Moreover, we validated the clinical role of LHCG-R score in EC cases. Indeed, we analyzed if the LHCG-R expression was related to local invasion and metastatic spread associated with grading (G3) and to lymphovascular space invasion (LVSI) [13].

## 2. Materials and Methods

### 2.1. Patients

Thirty (30) nonconsecutive EC hysterectomy specimens collected from April 2013 to October 2015 at Careggi University Hospital, Florence, Italy, were selected. Informed consent was obtained from each patient.

Inclusion criteria were type 1 EC (endometrioid subtype), grade 1 (G1) or 3 (G3), and availability of clinical data. The following parameters were recorded for each case: age, BMI (Body Mass Index), menopausal status, type of surgery, and FIGO stages. The histological classification was performed based on the World Health Organization (WHO) classification: grade 1 (G1) were endometrioid-type carcinomas composed of glands with <5% solid nonsquamous growth, while grade 3 (G3) had predominantly solid proliferation (>50%). Grade 2 (G2) cancers were intentionally excluded to identify two subgroups with different prognoses. The grading evaluation increases when nuclear atypia (large, pleomorphic nuclei, coarse chromatin, and large irregular nucleoli) was >50% in the tumor (WHO). LVSI was assessed for all patients.

All patients underwent surgery according to European Society of Gynecological Oncology (ESGO) guidelines. Only patients with presurgical stage ≥ IB underwent pelvic/para-aortic lymphadenectomy. Patients were staged retrospectively according to the 2009 FIGO staging guidelines. Patients defined as high-intermediate and high risk of recurrence according to ESGO guidelines [14] underwent adjuvant therapy.

Clinical, physiological, pathological, and follow-up features of each patient are reported in Supplementary Table 1.

EC hysterectomy specimens were fixed in formalin and embedded in paraffin (FFPE). Specific sections were performed and analyzed by hematoxylin-eosin staining and representative sections from each tumor were selected for IHC analysis.

### 2.2. Immunohistochemistry

IHC staining was performed on 3 *μ*m thick serial sections cut from formalin-fixed and paraffin-embedded (FFPE) tissues. The antibody used for the detection of LHCG-R was a rabbit, antihuman polyclonal antibody (Novus Biologicals, Littleton, Colorado, USA) at dilution 1 : 50, by overnight incubation at 4°C. Antigen retrieval was performed in a thermostatic bath (PT Link, Pretreatment Module, Dako, Agilent, Santa Clara, California, USA) at 97°C with Citrate buffer 10 mM pH 6 for 8 minutes. For chromogenic detection, ultraView Universal Alkaline Phosphatase Red Detection Kit (Ventana Medical Systems, Tucson, Arizona, USA) was used. The sections were lightly counterstained with Mayer's hematoxylin solution. A negative control sample was performed by omitting the primary antibody. Sections of corpus luteum were used as positive control. The control sections were treated with the samples in the same run.

### 2.3. Immunohistochemical (IHC) Scoring and Statistical Analysis

The IHC stained slides were microscopically analyzed by two independent observers (E.P., F.C.). Specimens were evaluated by focusing on the percentage of stained cells and the intensity of cytoplasm stain. The percentage of stained cells was graded as follows: 0 (0–24% of stained cells), 1 (25–49%), 2 (50–74%), and 3 (75–100%). The staining intensity was scored as follows: 0 (absent), 1 (weak), 2 (strong), and 3 (very strong). The sum of both parameters yielded the immunohistochemical (IHC) score, which ranged from 0 to 6 points. Tumors were divided into three categories depending on the IHC score: Category I corresponded to IHC score 0–2, Category II to IHC score 3-4, and Category III to a IHC score 5-6. Examples of IHC score and categories are shown in Supplementary Figure 1.

Chi-square statistics were used to test for correlations, the evaluation of Chi-square statistical significance was performed by *p* value. Statistical significance was considered achieved when the *p* value was less than or equal to 0.05. All statistical analyses were performed using Prism 7 for MAC.

## 3. Results and Discussion

### 3.1. Results

Thirty endometrioid EC tumors were evaluated: 15 (50%) G1 and 15 (50%) G3. The average age of the study population was 66 years (range: 43–81 years). The mean BMI was 31 (range: 19.5–40). Most of the patients were in menopause (97%) and only one was in premenopause (3%); the average menopausal age was 52.4 years (range: 46–59 years). Eleven patients underwent lymphadenectomy (11/30; 36.6%); among these patients, there was only one case with node invasion (9%). Thirteen patients (13/30; 43.3%) underwent adjuvant therapy: 2 patients had combined chemo- and radiotherapy (15%) and 11 patients had only radiotherapy (85%). Patients had an average follow-up of 30 months (range: 18–49 months); during the study period one patient died of disease (DOD) after 15 months of follow-up (FU) and one patient experienced nodal recurrence at 22 months of FU and she is still alive. Supplementary Table 1 showed patients' clinical data and FU.

According to the IHC score, 11 patients (11/30; 36.6%) were classified in IHC score category I, 9 patients (9/30; 30%) in category II, and 10 patients (10/30; 33.3%) in category III. The agreement between the two observers was 95%.

No significant distribution was observed among IHC score categories and clinical, physiological, and pathological features (BMI, menopause, myometrial invasion, FIGO stage, presence of distant recurrence, FU, and survival) except for grading and LVSI.

**Figure 1:**
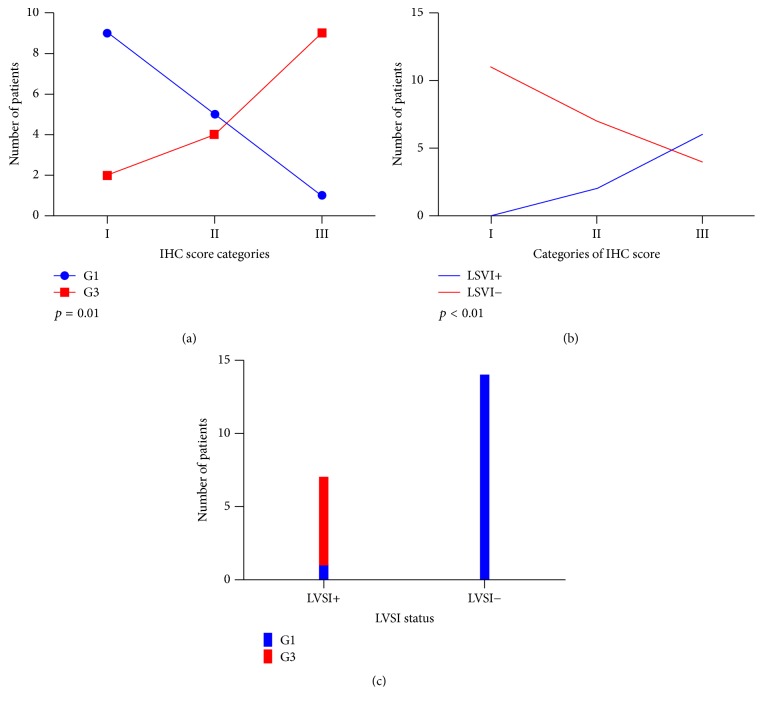
Correlation between LHCG-R IHC score categories and pathological features. (a) LHCG-R IHC score categories and grading (G1 and G3). (b) LHCG-R IHC score categories and LVSI. LVSI+: positive LVSI. LVSI−: negative LVSI. (c) Correlation between LVSI (LVSI+ and LVSI−) and grading (G1 and G3).

Regarding the correlation between IHC score category and grading, within IHC score category I, 9 patients (9/11; 81.8%) were G1 and 2 (2/11; 18.18%) were G3; within IHC score category II, 5 patients (5/9; 55.5%) were G1 and 4 (4/9; 44.4%) were G3; finally, within IHC score category III there was only one G1 patient (1/10; 10%) and 9 G3 patients (9/10; 90%). This distribution showed a statistically significant positive relation between grading and IHC scoring (*p* = 0.01) (Figure 1(a)).

Concerning the LVSI, 8 patients (8/30; 26.6%) presented LVSI and 22 (22/30; 73.3%) were without LVSI. Within the LVSI-positive patients, 2 (2/8; 25%) were in IHC score category II and 6 (6/8; 75%) in IHC score category III. Within LVSI-negative patients, there were 11 patients (11/22; 50%) in IHC score category I, 7 (7/22; 31.8%) in category II, and 4 (4/22; 18.2%) in category III (Figure 1(b)). There was a statistically significant positive relation between LVSI and IHC score (*p* < 0.01). Notably, among the 8 LVSI-positive patients, 7 (7/8; 87.5%) were G3 and 1 (1/8; 12.5%) was G1 (*p* = 0.035) (Figure 1(c)).

Finally, both the patients with nodal recurrence and DOD were in IHC score category III.

## 4. Discussion

Endometrial cancer (EC) is the fourth most common cancer overall and the most common malignancy of the female reproductive tract and its incidence is increasing [15]. Several epidemiological and histological factors such as increasing age, depth of myometrial invasion, histological tumor type and grade, presence of LVSI, and FIGO stage have been reported to be correlated with a higher risk of recurrence and nodal metastases in early-stage EC [14, 16–18]. Nevertheless, the recurrence rate in these patients is widely variable, ranging from 2% to 26% [16, 17, 19].

Therefore, not only is the identification of key factors/pathways responsible for the aggressiveness of EC, but also additional prognostic tools are urgently needed to improve the definition of a patient's risk of recurrence [15].

Several biomarkers are under investigation for prognostic, diagnostic, or therapeutic aims in EC, though no one of them is currently used in clinical practice [14]. Numerous studies were focused on the research of biomarkers for discrimination of type I from type II mainly based on mRNA expression in tissues [20–22] or on IHC procedure (i.e., L1CAM, PR, ER, STMN, and PTEN, USP14, Ki-67, even if the latest is not a specific biomarker for EC, it is frequently and routinely used for several kinds of tumors) [23–26] or in biological fluid [27]. The most used biomarker is circulating CA 125 but not only is the sensitivity unsatisfactory, especially in early tumor stage, but also its specificity is low. Nevertheless CA125 has no correlation with the prognosis of the patient [28].

Recently, IHC scores based on the evaluation of ER and HER-2 staining [29, 30] and PTEN were described [31]. Moreover, specific scores for prediction of recurrence have been set based on gene signature and IHC [32].

The presence and role of the LH/hCG receptor (LHCG-R) in EC have already been described in previous studies by our group [6–10]. Indeed, our group has already demonstrated that (i) LH/hCG induces an in vitro invasive phenotype, through the activation of LHCG-R and hence of PKA [6]; (ii) LHCG-R mRNA is expressed in a small cohort primary ECs [7]; (iii) primary treatment with Gn-RH analogues (aimed to decrease the levels of serum LH) for six years in a patient affected by EC with contraindications to surgery was associated with no evidence of progression of the disease throughout the study period [9]; and (iv) LHCG-R in a EC preclinical mouse model behaves such as a prometastatic molecular device [8]. Finally, we recently published a case of a 51-year-old affected by G2 endometrioid EC, FIGO stage IA. According to the conventional prognostic factors, the patient was treated as a low-risk EC with surgery alone and close follow-up. Surprisingly, six months after surgery she developed an early vesicovaginal recurrence and, a few months later, a subsequent involvement of the vaginal wall, ileum, and omentum, despite the chemotherapy. We determined the LHCG-R expression in the surgical samples both at mRNA level and at protein level; both evaluations turned out to be highly expressed [9]. The previously published data, together with those presented in this manuscript, may suggest a relation between LHCG-R expression and poor prognosis in EC.

In this manuscript, we focused on the evaluation of prognostic use of LHCG-R in a selected subgroup of sample characterized by type I EC in order to investigate receptor level and unexpected recurrences.

The presence of LHCG-R was investigated by IHC and we set a score that considers the amount (intensity) and spread (percentage of stained cells) of LHCG-R positive cells. Even if a small cohort was analyzed, we showed a correlation between LHCG-R IHC score and LVSI and Grading. G3 patients have a significantly higher score than G1 patients; similarly, LVSI-positive patients have a significantly higher score than LVSI-negative patients, suggesting a key role played by this receptor in EC cancer development in terms of invasiveness and bad prognosis. Moreover, the evaluation of LHCG-R expression can be used for diagnostic purpose, but also for treatment setting. In a previous study [9], we noticed that the treatment in a patient presenting high level of LHCG-R and the use of LHGC-R analogues allowed maintaining the tumor without increasing of growth and invasion.

All these data point out the fact that the evaluation of LHGC-R could be adopted as a new biomarker for implementing decision-making process of the pathologist and overcoming the pitfalls harbored in the grading and LVSI assessment for some critical samples. Therefore, the LHCG-R IHC score might represent a new tool to better identify EC patients with negative prognostic factors.

## Supplementary Materials

Supplementary 1 Supplemental Table 1: clinical physiological and pathological characteristics and follow-up of patients. y: years; BMI: body mass index; ECa: endometrioid EC; m: months; NED: no evidence of disease; DOD: died of disease; FU: follow-up.

Supplementary 2 Supplemental Figure 1: LHCG-R IHC score evaluation in endometrial cancer: two example cases. Panels A and B: hematoxylin-eosin staining; panels C and D: corresponding immunohistochemistry (IHC) for LH/hCG-R. Panel A (patient 1): endometrioid adenocarcinoma grade 1 composed of atypical glands (arrow head) with nuclear atypia (magnification X20). Panel B (patient 2): endometrioid adenocarcinoma with more than 50% of a nonsquamous or nonmorular solid growth pattern (star) (magnification, X20). Panel C (patient 1): LHCG-R IHC with very strong intensity at cytoplasmatic level (score 3) and 80 % of cells stained (score 3), LHCG-R IHC score category III (magnification X20). Panel D (patient 2): some areas with strong intensity and others with weak or absent (arrow) stained cells (score 2) and about 60 % of stained cells (score 2), LHCG-R IHC score category II (magnification X20).
