# Supplementary material for: Luteinizing Hormone/Human Chorionic Gonadotropin Receptor Immunohistochemical Score Associated with Poor Prognosis in Endometrial Cancer Patients
Source: Biomed Res Int. 2018 Apr 2;2018:1618056. doi: 10.1155/2018/1618056 (PMC5902075; doi:10.1155/2018/1618056)
Supplement: Supplementary 1 — Supplemental Table 1: clinical physiological and pathological characteristics and follow-up of patients. y: years; BMI: body mass index; ECa: endometrioid EC; m: months; NED: no evidence of disease; DOD: died of disease; FU: follow-up. [file 1618056.f1.docx]

| Patient age (y) | BMI | Menopause age (y) | Histology | Myometrial invasion | FIGO stage | Grading | FU status | FU (m) |
| --- | --- | --- | --- | --- | --- | --- | --- | --- |
| 57 | 29.08 | 53 | ECa | >50% | I B | G3 | nodal recurrence at 22 m of FU | 49 |
| 61 | 29.45 | 59 | ECa | <50% | I A | G1 | NED | 47 |
| 75 | 24.75 | 46 | ECa | >50% | I B | G3 | NED | 47 |
| 54 | 25.37 | 49 | ECa | <50% | I A | G1 | NED | 47 |
| 62 | 35.37 | 47 | ECa | <50% | I A | G1 | NED | 48 |
| 76 | 23.76 | 54 | ECa | <50% | I A | G1 | NED | 41 |
| 66 | 26.95 | 56 | ECa | <50% | I A | G1 | DOD | 15 |
| 58 | 31.25 | 55 | ECa | <50% | I A | G1 | NED | 33 |
| 53 | 24.91 | 51 | ECa | <50% | I A | G1 | NED | 32 |
| 65 | 35.16 | 50 | ECa | >50% | III C1 | G3 | NED | 32 |
| 78 | 37.11 | 55 | ECa | >50% | I B | G3 | NED | 29 |
| 67 | 22.8 | 50 | ECa | <50% | I A | G1 | NED | 28 |
| 44 | 34.72 | 52 | ECa | <50% | I A | G1 | NED | 25 |
| 61 | 26.48 | 52 | ECa | >50% | I B | G3 | NED | 26 |
| 57 | 32.43 | 53 | ECa | <50% | I A | G1 | NED | 29 |
| 55 | 19.5 | 52 | ECa | <50% | I A | G1 | NED | 27 |
| 69 | 38.7 | 52 | ECa | >50% | III A | G3 | NED | 26 |
| 55 | 22.22 | 54 | ECa | >50% | I B | G3 | NED | 25 |
| 76 | 23.91 | 55 | ECa | >50% | I B | G1 | NED | 27 |
| 72 | 33.2 | 52 | ECa | >50% | II | G3 | NED | 28 |
| 81 | 22 | 50 | ECa | >50% | I B | G3 | NED | 29 |
| 75 | 23.2 | 56 | ECa | <50% | I A | G1 | NED | 24 |
| 81 | 40 | 52 | ECa | >50% | I B | G3 | NED | 28 |
| 80 | 31.25 | 50 | ECa | >50% | II | G3 | NED | 29 |
| 77 | 23.05 | 53 | ECa | >50% | I B | G3 | NED | 22 |
| 43 | 21.92 | - | ECa | <50% | I A | G3 | NED | 31 |
| 79 | 39.06 | 50 | ECa | <50% | I A | G1 | NED | 19 |
| 74 | 36 | 54 | ECa | <50% | I A | G3 | NED | 19 |
| 66 | 31.25 | 53 | ECa | <50% | I A | G1 | NED | 20 |
| 69 | 26 | 56 | ECa | <50% | I A | G3 | NED | 18 |
