# Supplementary figures and images for: Luteinizing Hormone/Human Chorionic Gonadotropin Receptor Immunohistochemical Score Associated with Poor Prognosis in Endometrial Cancer Patients
Source: Biomed Res Int. 2018 Apr 2;2018:1618056. doi: 10.1155/2018/1618056 (PMC5902075; doi:10.1155/2018/1618056)

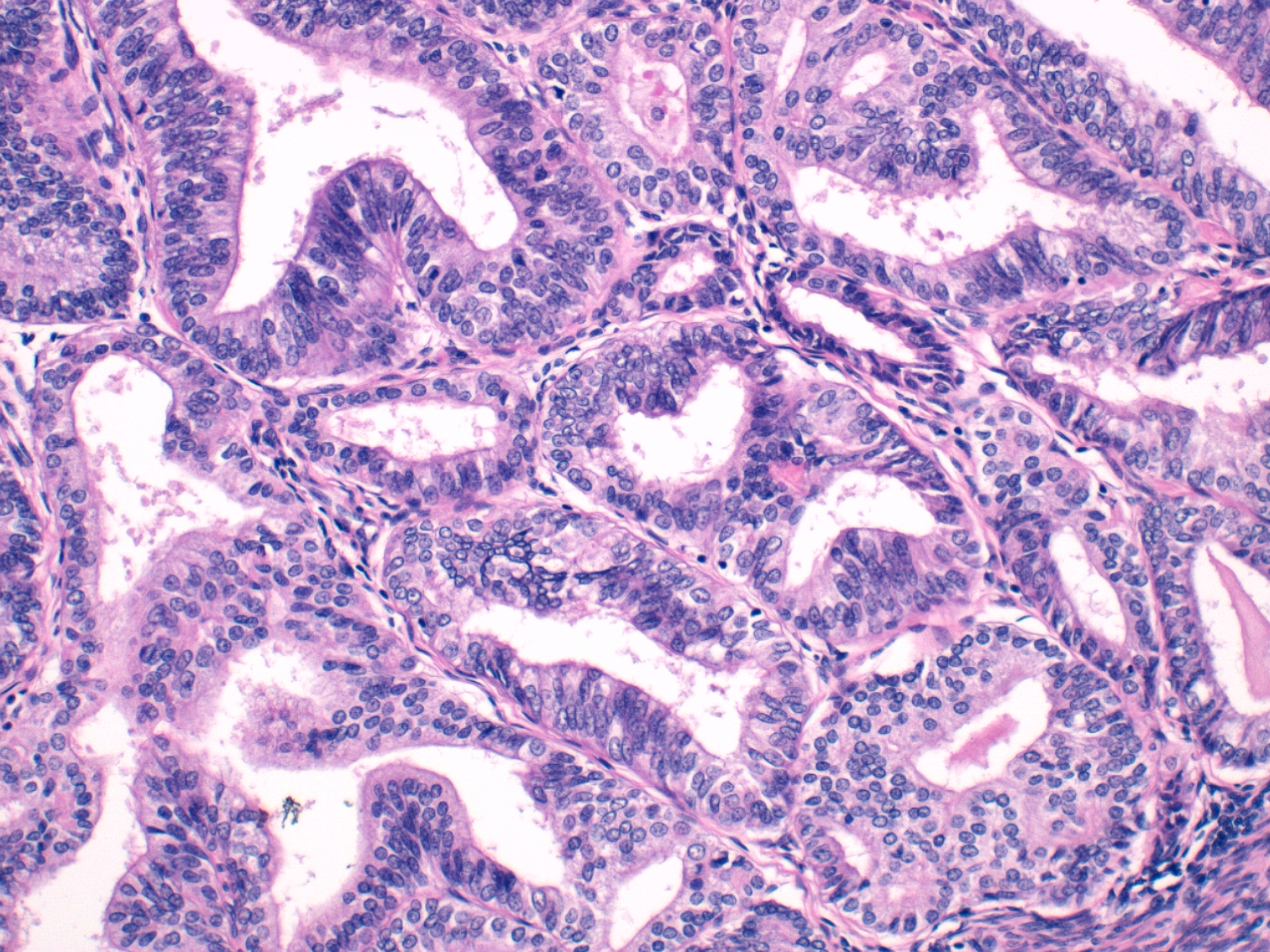

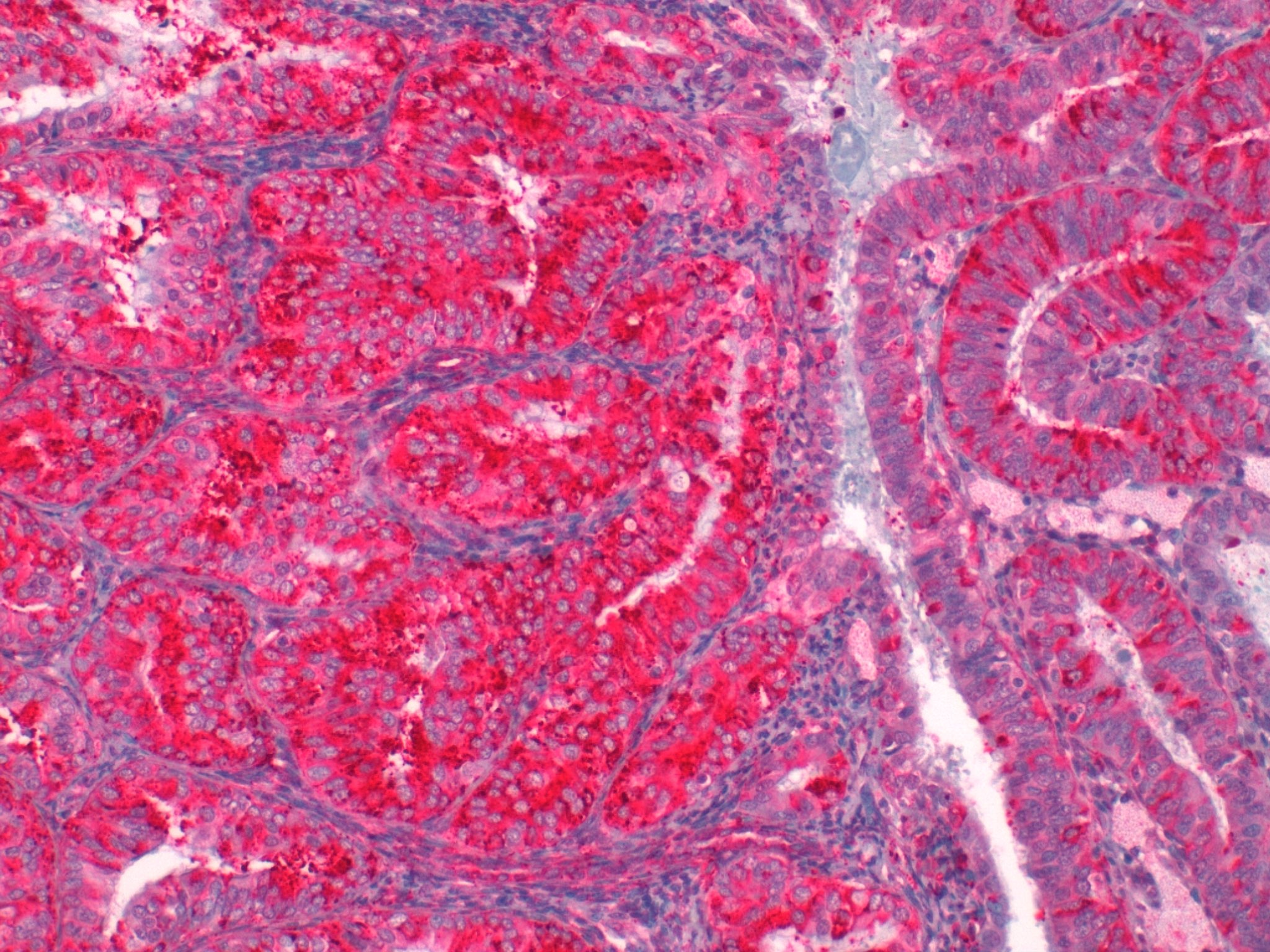

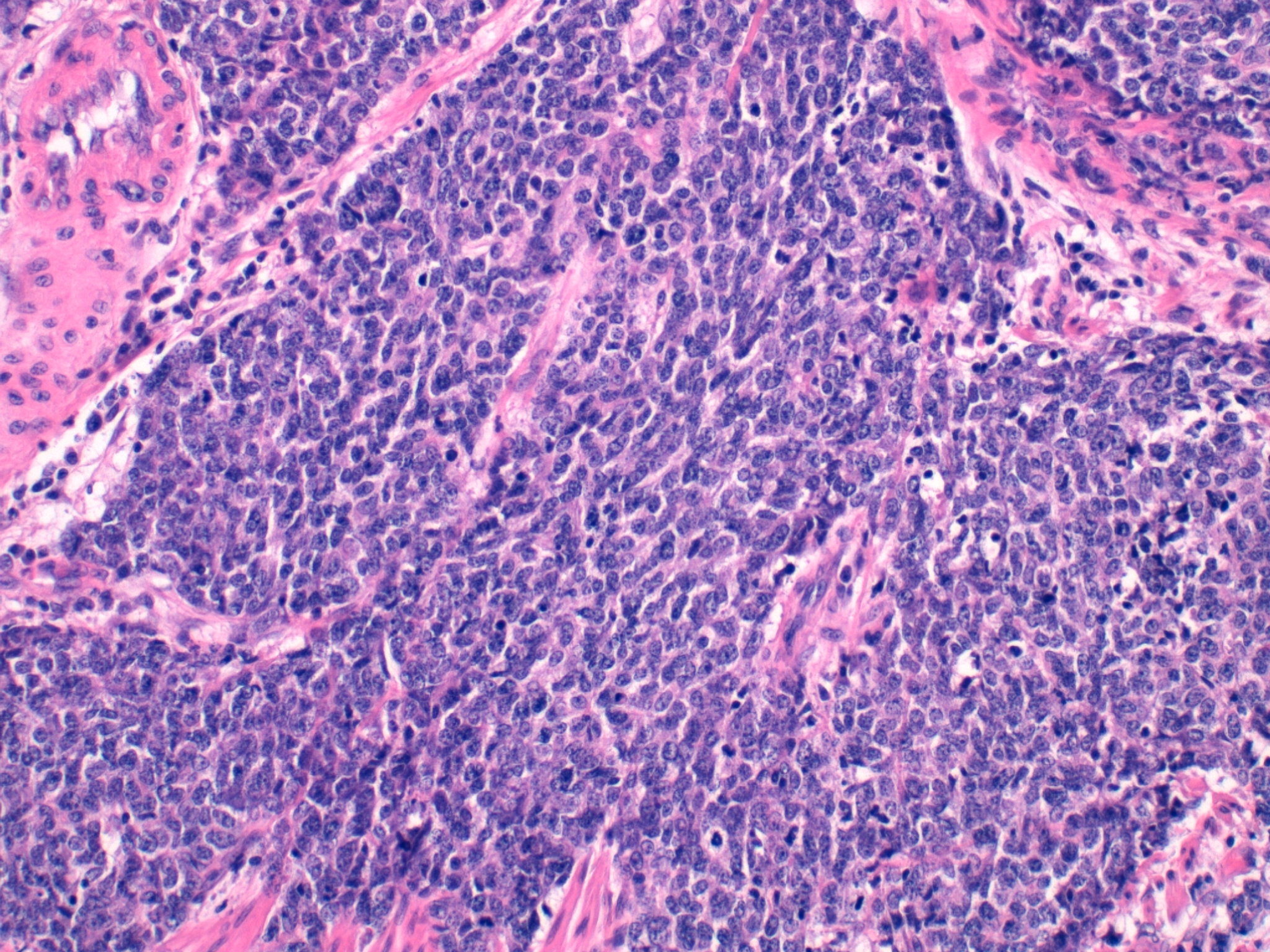

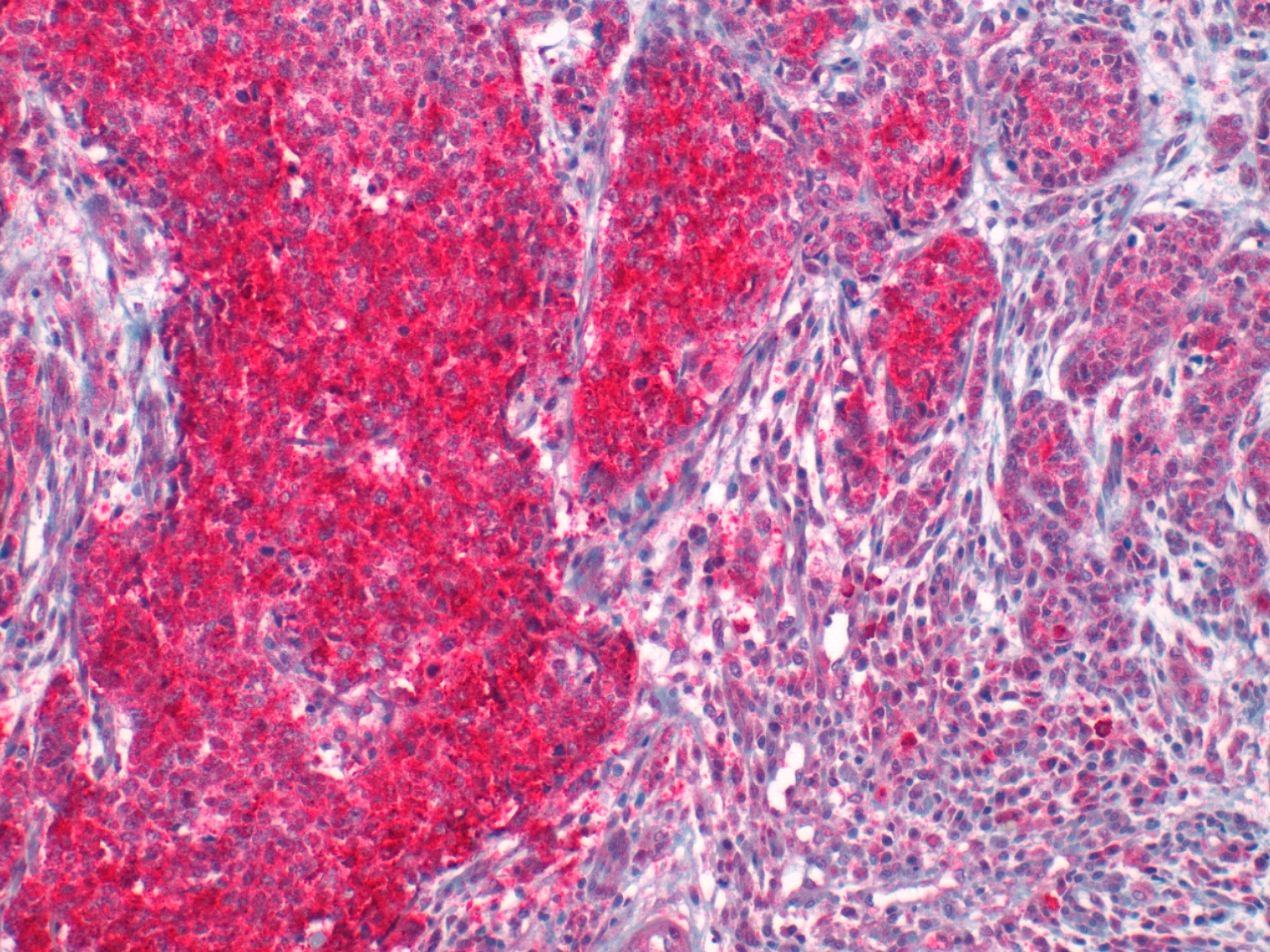


A

C

B

D

Supplement: Supplementary 2 — Supplemental Figure 1: LHCG-R IHC score evaluation in endometrial cancer: two example cases. Panels A and B: hematoxylin-eosin staining; panels C and D: corresponding immunohistochemistry (IHC) for LH/hCG-R. Panel A (patient 1): endometrioid adenocarcinoma grade 1 composed of atypical glands (arrow head) with nuclear atypia (magnification X20). Panel B (patient 2): endometrioid adenocarcinoma with more than 50% of a nonsquamous or nonmorular solid growth pattern (star) (magnification, X20). Panel C (patient 1): LHCG-R IHC with very strong intensity at cytoplasmatic level (score 3) and 80 % of cells stained (score 3), LHCG-R IHC score category III (magnification X20). Panel D (patient 2): some areas with strong intensity and others with weak or absent (arrow) stained cells (score 2) and about 60 % of stained cells (score 2), LHCG-R IHC score category II (magnification X20). [file 1618056.f2.docx]
